# Bridging to Transplant: TDM-guided Outpatient Dalbavancin Therapy in Chronic Granulomatous Disease With Deep-seeded Inoperable Abscesses Over 11 Months

**DOI:** 10.1093/ofid/ofaf260

**Published:** 2025-04-30

**Authors:** Micha Banz, Stefan Hagel, Fritz Sörgel, Martina Kinzig, Katrin Farker, Mathias W Pletz, Karim Kentouche

**Affiliations:** Department of Gastroenterology, Hepatology, and Infectious Diseases, Clinic of Internal Medicine IV, Jena University Hospital, Jena, Germany; Institute for Infectious Diseases and Infection Control, Jena University Hospital, Jena, Germany; Institute for Infectious Diseases and Infection Control, Jena University Hospital, Jena, Germany; IBMP-Institute for Biomedical and Pharmaceutical Research, Nürnberg-Heroldsberg, Germany; Institute of Pharmacology, West German Heart and Vascular Centre, University of Duisburg-Essen, Essen, Germany; IBMP-Institute for Biomedical and Pharmaceutical Research, Nürnberg-Heroldsberg, Germany; Hospital Pharmacy, University Center for Pharmacotherapy and Pharmacoeconomics, Jena University Hospital, Jena, Germany; Institute for Infectious Diseases and Infection Control, Jena University Hospital, Jena, Germany; Department of Paediatrics, Jena University Hospital, Jena, Germany

**Keywords:** chronic granulomatous disease, dalbavancin, mixed bacterial abscesses, outpatient parenteral antimicrobial therapy, therapeutic drug monitoring

## Abstract

Therapeutic drug monitoring facilitated the successful outpatient use of low-dosage dalbavancin for treating inoperable staphylococcal abscesses in a patient with chronic granulomatous disease. This approach minimized adverse effects and allowed for effective infection control, highlighting its potential role in complex infectious cases requiring long-term antimicrobial therapy.

## Teaser

We present the case of a young male patient with familial chronic granulomatous disease who developed subcutaneous abscesses and fistulas in the inguinal and retroperitoneal regions, caused by coagulase-negative staphylococci and *Acinetobacter lwoffii*. The complexity and extent of the abscesses rendered surgical drainage unfeasible. As a result, an extended 11-month course of dalbavancin therapy, far exceeding the standard approved duration, was initiated and guided by therapeutic drug monitoring. This approach allowed for dose optimization, reducing the dalbavancin dosage to 500 mg every 2 weeks, leading to significant clinical improvement and a recovery from the abscesses. This report highlights the innovative application of dalbavancin with therapeutic drug monitoring in an outpatient setting for the management of gram-positive bacterial infections in soft tissue, demonstrating its potential benefits in terms of safety, cost-effectiveness, and the ability to treat patients outside the hospital.

Patients with chronic granulomatous disease (CGD) are susceptible to infections by catalase-positive organisms, including *Staphylococcus* spp., *Burkholderia cepacia complex, Serratia marcescens, Nocardia*, and *Aspergillus* species that can cause infections in the lungs, lymph nodes, skin, liver, and bones, respectively. Most common causes of infection-related mortality in patients with CGD in Europe are by fungal infections by *Aspergillus* or more rarely by *Candida* species [1].

Infectious diseases in patients with CGD often result in recurrent, severe infections with a high probability of chronification because of abscess formation in the tissue and accumulation of phagocytes at the site of inflammation. The underlying condition weakens the host response by depletion of granulocytic cells, making the host incapable of warding off pathogens with granulocytic oxidative bursts [2]. These cells typically encapsulate invading pathogens at the site of infection, forming characteristic granulomas and abscesses. Until today, treatment of acute infections in patients with CGD has been challenging because of the increased probability of encapsulation in abscesses, as well as the overall reduction of host defense mechanisms, even in bacteria showing high sensitivity to antibiotics. Treatment often consists of an aggressive and prolonged therapy regimen of antimicrobial agents that penetrate deep into the infected tissue [3].

## CASE REPORT

In 2021, a 23-year-old male with X-linked CGD (hemizygote large deletion in the *CYBB* gene) was evaluated for routine care. COVID-19 lockdowns had delayed his check-ups by 15 months. The patient reported a weight loss of 8 kg over the months before presentation. The patient's regular medication included co-trimoxazole 960 mg (800 mg sulfamethoxazole/160 mg trimethoprim) and itraconazole 100 mg, each taken twice daily, and ramipril 5 mg once daily for essential hypertension.

At presentation, the patient weighed 71 kg and had normal renal function, with an estimated glomerular filtration rate of >100 mL/min/1.73 m^2^.

**Figure 1.**
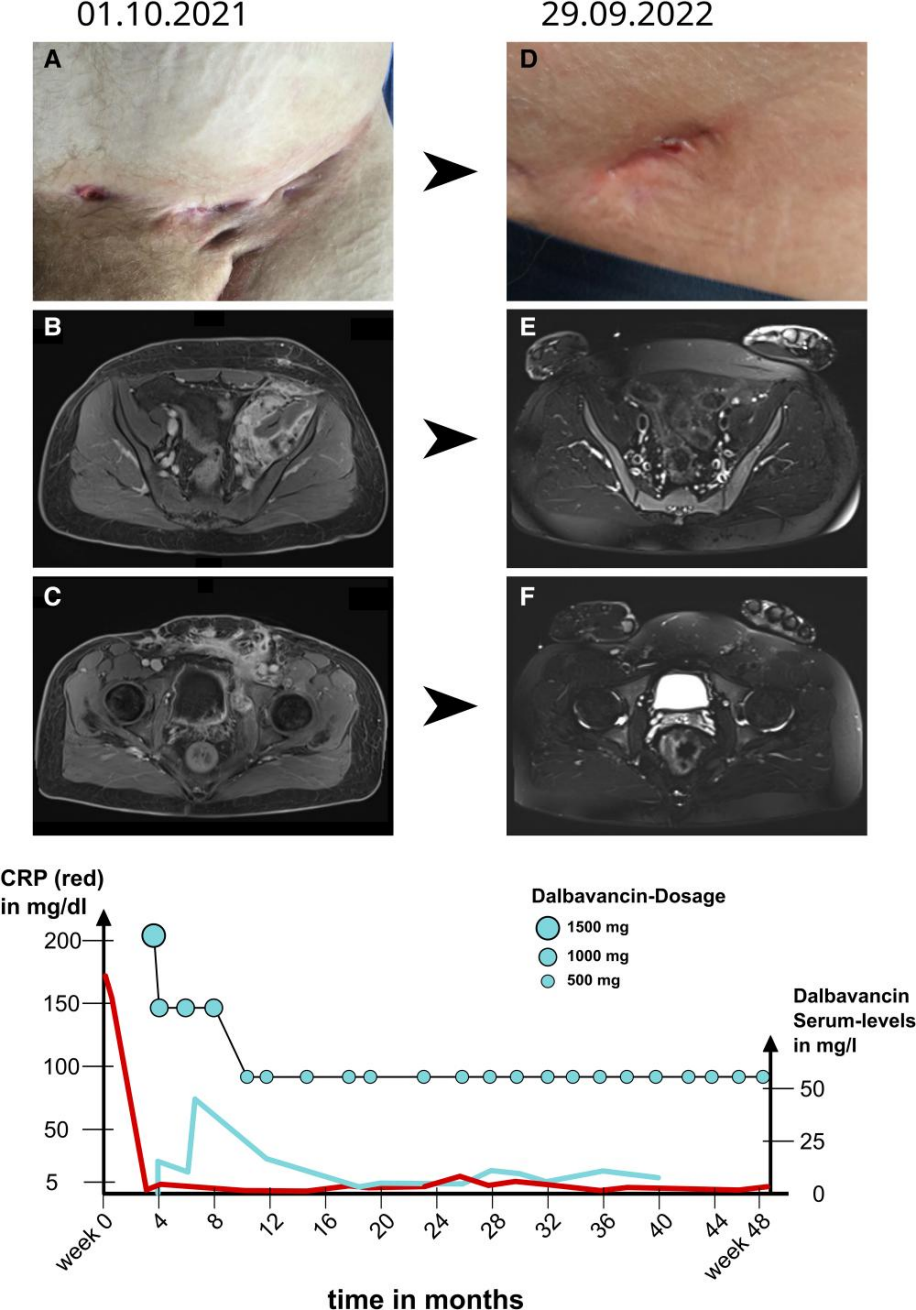
Images of inguinal abscesses and magnetic resonance imaging (MRI) over the course of the treatment and graphical treatment overview. *A–C*: Pretreatment clinical photograph and MRI scans showing multiple deep abscesses in the inguinal region, extending toward the sacrum, bladder, and intestines. *D–F*: Posttreatment images showing significant resolution of abscesses following dalbavancin therapy, with only 1 external remnant remaining. MRI scans show no residual abscesses. Graph: Schematic of C-reactive protein (CRP) and dalbavancin serum levels over time, aligned with the biweekly dosing. Turquoise dots, sized according to the administered amount, indicate the timing and dosage of dalbavancin.

During the clinical examination, multiple pus-discharging abscesses were observed in the inguinal region (Figure 1). Elevated inflammatory markers and magnetic resonance imaging indicated an extensive tissue infection with fistulous tracts extending to the os sacrum, superficially affecting the bladder and bowel. He was admitted for treatment with intravenous meropenem, teicoplanin, and posaconazole. Microbiological cultures, obtained from deep wound swabs, revealed a polymicrobial infection, including 2 different strains of coagulase-negative staphylococci (CoNS) not further specified and *Acinetobacter lwoffii*. One of the CoNS strains showed resistance to macrolides, oxacillin, fosfomycin, fluoroquinolones, co-trimoxazole, and gentamicin. One of the CoNS strains showed resistance to macrolides, oxacillin, fosfomycin, fluoroquinolones, co-trimoxazole, gentamicin, and doxycycline, ruling out tetracyclines as a treatment option. In both strains, a minimal inhibitory concentration (MIC) of 1.0 mg/L for vancomycin was reported. Alongside these, an *A lwoffii* strain was detected, which was tested with susceptibility against meropenem, co-trimoxazole, gentamicin, tobramycin, and tigecycline. Ciprofloxacin susceptibility was tested and categorized as intermediate, allowing for its oral use in the outpatient setting.

Because of extensive tissue and bone infection a surgical source control seemed not possible; hence, a long-term antibiotic therapy with ciprofloxacin and dalbavancin was initiated by regular intravenous infusions as an outpatient. Alternative glycopeptides such as vancomycin, teicoplanin, or telavancin were not selected due to the need for central venous access and their less favorable side effect profiles in long-term use. In this patient, outpatient therapy without central line placement was preferred because of infection risk, logistical challenges, and the extended treatment duration. Dalbavancin was commenced with an initial dose of 1500 mg, followed by 3× 1000 mg every 2 weeks. A key advantage of dalbavancin in this case was that it could be administered without the need for a central venous catheter, avoiding the risks of catheter-associated infections and enabling safe, long-term outpatient therapy. At every date of intravenous treatment, blood was collected and sent to a collaborator for quantification of dalbavancin levels using liquid chromatography mass spectrometry (LC-MS). The average turnaround time for results was 3 days, which allowed for dose adjustments during ongoing therapy planning. Although dalbavancin infusions were scheduled in advance at regular intervals, serum levels were used retrospectively to inform dose adjustments for subsequent infusions. In 1 instance, after a trough concentration of 7.73 µg/mL, an additional dose was scheduled earlier than planned to maintain therapeutic levels. Using the results of LC-MS quantification, while aiming for a trough serum concentration of dalbavancin >8.04 mg/L, dosing of dalbavancin could be reduced to 500 mg per infusion, guided by therapeutic drug monitoring (refer to the schematic overview in Figure 1 and the detailed data in extended Table 1).

**Table 1.** Dalbavancin Dosing and Trough Levels: Administered Dose (mg) and Corresponding Serum Concentration (µg/mL) Measured by Liquid Chromatography Mass Spectrometry Over the 11-month Treatment Period

Sampling Date	Administered Dose (mg)	Serum Concentration (µg/mL)
Week 0	…	…
Week 1	…	…
Week 3	1.500	159
Week 4	1.000	26.23
Week 6	1.000	19.06
Week 7	…	74.88
Week 8	1.000	…
Week 10	500	…
Week 12	500	27.1
Week 15	500	…
Week 19	500	…
Week 20	500	9.06
Week 23	500	8.03
Week 26	500	9.39
Week 28	500	18.6
Week 30	500	15.7
Week 32	500	11.1
Week 34	500	16.3
Week 36	500	19.2
Week 38	500	14.7
Week 40	500	13.6
Week 42	500	…
Week 44	500	…
Week 46	500	…
Week 48	500	…

Last serum concentration was measured in week 40.

The patient's skin and soft tissue infection responded well to the antibiotic regimen, with the patient not reporting any adverse events related to antibiotic therapy (Figure 1). Dalbavancin therapy was stopped after overall 48 weeks of therapy and bone marrow transplantation was reconsidered. A 10/10 human leukocyte antigen–matched unrelated donor was available and preparation for bone marrow transplantation in late November 2022 followed a reduced intensity conditioning protocol with targeted busulfan administration [4], leading to only minor complications. Before transplantation, the patient received a prophylactic antibiotic regimen including ciprofloxacin 750 mg 1-0-1, co-trimoxazole 960 mg 1-0-1, and itraconazole 100 mg 1-0-1. During aplasia, no relapse of infection was noticed.

In the posttransplantation phase, the patient developed autoimmune cytopenia and recovered gradually. He demonstrated a stable mixed chimerism with 100% donor granulocytes and no signs of graft-versus-host disease, allowing for a gradual return to daily activities. While hospitalized and during outpatient follow-up, frequent microbiological surveillance of skin and wound sites revealed no development of resistance in either commensal or pathogenic bacteria. No new pathogens were cultured and repeat swabs of residual fistulas showed persistence of the initial flora without new resistance traits.

## DISCUSSION

CGD is a hereditary immunodeficiency disorder, characterized by mutations in various genes, coding for NADPH oxidases. These mutations lead to a defective oxidative burst in phagocytes, thereby impairing the body's defense against specific bacteria and fungi. Besides tackling the infections, the only curative treatment of CGD consists of the transplantation of hematopoietic stem cells [5]. Advances in antimicrobial therapies, like azole antifungals, have improved life expectancy significantly, with a median of 37.8 years for X-linked patients and 49.6 years for autosomal recessive cases [6]. However, infection management remains complex, often requiring long-term, aggressive antimicrobial regimens [7].

Dalbavancin, a lipoglycopeptide with a favorable safety profile, was chosen for its ability to achieve high tissue penetration with minimal side effects, offering a more manageable outpatient treatment option compared to alternatives like linezolid, which can cause bone marrow suppression, or clindamycin, associated with *Clostridium difficile* infection.

Dalbavancin is distinguished by its notably low incidence of adverse effects, a significant advantage over the limited alternatives available. In this case, potential adverse events were actively monitored and solicited during follow-up visits, but none was reported by the patient throughout the 11-month treatment period. Despite the drawback of its high cost, with infusions priced at ∼€2000 per gram, the use of dalbavancin proved to be economically favorable in retrospect, compared to the substantial expenses associated with prolonged inpatient care necessitating intravenous antibiotics.

Dalbavancin is specifically well-suited for outpatient treatment because of its long half-life, which allows for infrequent dosing—typically every 1 to 2 weeks as have been shown in the Dalbavancin as an option for treatment of *S. aureus* bacteremia (DOTS) trial [8]. Contrary to the DOTS trial protocol, rather than administering 2 doses of 1500 mg at 2 time points, we monitored the trough levels through TDM using high-performance liquid chromatography (HPLC) to measure serum dalbavancin concentrations. Based on these results, we were able to reduce both the dose and the interval to 500 mg every 2 weeks over an extended period. In this case, TDM played a crucial role in optimizing treatment by ensuring that appropriate drug levels were maintained [8]. In contrast to other recent TDM-guided protocols that extended dosing intervals beyond 4 weeks [9, 10], we opted to maintain a biweekly schedule and reduce the dose instead. This decision also facilitated regular clinical follow-up during the off-label use of dalbavancin, ensuring patient safety and close monitoring throughout the prolonged treatment course.

Despite increasing clinical experience, long-acting glycopeptides such as dalbavancin are not yet formally included in major clinical guidelines for long-term treatment of deep tissue infections. Their use in this context remains off-label and based on real-world data and expert consensus. This approach minimized the risk of adverse effects and maximized cost-effectiveness by avoiding unnecessary overdosing, further enhancing the benefits of outpatient care with dalbavancin.

During the outpatient administration of dalbavancin, we closely monitored serum drug levels via HPLC, which enabled us to adjust the dosage to 500 mg per infusion cycle [11]. For sustained treatment, achieving dalbavancin trough serum concentrations greater than 8 mg/L is deemed efficacious, particularly in cases involving *Staphylococcus* spp. infections [12]. A dalbavancin MIC was not determined; instead, we guided therapeutic monitoring based on a vancomycin MIC of 1 mg/L for both CoNS isolates, consistent with established pharmacokinetics/pharmacodynamics conversion models [13]. Furthermore, the use of TDM provided critical insights into the pharmacokinetics of dalbavancin in a real-world clinical setting, highlighting the drug's ability to maintain effective concentrations in tissue over extended periods. By adapting the dosing regimen, the medical team was able to control the infection, leading to significant regression in the size and number of abscesses, all while avoiding hospital admission.

The use of TDM in this case, specifically for dose adjustment, underscores the value of establishing a diagnostic workflow in clinical chemistry laboratories—particularly in infectious disease departments—that includes HPLC for dalbavancin monitoring. This practice should not be limited to rare or refractory cases. Many more infectious diseases, such as bone infections, joint infections, endocarditis, or other abscess-forming deep tissue infections, could benefit from outpatient dalbavancin use, as is already being done successfully in the United States [14]. TDM enables true individualized medicine by tailoring antibiotic exposure to patient-specific pharmacokinetics, thereby reducing both morbidity and unnecessary toxicity. Crucially, TDM also makes long-term outpatient care with expensive agents like dalbavancin economically feasible. In this case, avoiding prolonged inpatient treatment and central line placement—supported by safe, reduced dosing—led to a significant cost advantage. As the cost of LC-MS–based TDM is minor compared to hospitalization, its broader integration into infectious disease care could reduce overall healthcare system burden while improving outcomes.

## CONCLUSION

This case demonstrates the potential of outpatient parenteral antibiotic therapy, guided by TDM, in managing complex infections in immunocompromised patients. Dalbavancin, selected for its efficacy against gram-positive bacteria and long half-life, allowed for reduced dosing and minimized the need for frequent intravenous access. The successful use of TDM to adjust dosing helped resolve inoperable staphylococcal abscesses, supporting the long-term management of infection-related complications. Notably, there were no adverse drug effects reported, making the use of dalbavancin in this case safe to use on a long-term basis. Also, frequent microbiological testing showed no sign of resistance development in commensal bacteria. Following treatment, the patient underwent a successful bone marrow transplantation without relapse during the aplasia phase.

## Notes

***Previous presentation*.** The information in this manuscript has not been presented at any meeting or conference.

***Patient consent statement*.** Informed consent from the patient was secured.
